# A promising new predictive factor for detecting bowel resection in childhood intussusception: the lymphocyte-C-reactive protein ratio

**DOI:** 10.1186/s12887-021-03068-2

**Published:** 2021-12-16

**Authors:** Bailin Chen, Jian Cao, Chengwei Yan, Chao Zheng, Jingyu Chen, Chunbao Guo

**Affiliations:** 1 Department of Pediatric General Surgery, Children’s Hospital of Chongqing Medical University, Chongqing, People’s Republic of China; 2 Ministry of Education Key Laboratory of Child Development and Disorders, National Clinical Research Center for Child Health and Disorders, China International Science and Technology Cooperation base of Child development and Critical Disorders, Chongqing Key Laboratory of Pediatrics, Children’s Hospital of Chongqing Medical University, Chongqing, People’s Republic of China; 3 Department of Pediatric General Surgery, Chongqing University Three Gorges Hospital, Chongqing, People’s Republic of China; 4 Department II of Orthopedics, Children’s Hospital of Chongqing Medical University, 136 Zhongshan 2nd Rd., Chongqing, 400014 People’s Republic of China; 5 Department of Ultrasound, Children’s Hospital of Chongqing Medical University, 136 Zhongshan 2nd Rd., Chongqing, 400014 People’s Republic of China

**Keywords:** Intussusception, Lymphocyte–CRP ratio, Intestinal resection, Intestinal ischemia

## Abstract

**Background:**

The most critical concern for the management of childhood intussusception is bowel resection due to intestinal ischemia and necrosis. The early prediction of this problem is of great importance. We investigated the value of various combinations of inflammatory factors to predict intestinal necrosis and resection.

**Methods:**

We retrospectively reviewed the medical records of pediatric patients with intussusception who underwent surgical management. During the research period, 47 patients who underwent intestinal resection due to intestinal necrosis and 68 patients who did not undergo intestinal resection were enrolled. We evaluated the diagnostic value of various combinations of inflammatory markers from preoperative laboratory analyses using the receiver operating characteristic (ROC) method.

**Results:**

In the current cohort, 115 patients underwent operations for intussusception; among them, 47 patients (40.9%) underwent intestinal resections. In the patients with intestinal resection, the neutrophil count(*p* = 0.013), CRP level(*p* = 0.002), platelet–lymphocyte ratio (PLR, *p* = 0.008), NLR (neutrophil–lymphocyte ratio, *p* = 0.026), and LCR (lymphocyte–CRP ratio, *p* < 0.001) values were significantly higher than those in the patients without any resection. The receiver operating characteristic (ROC) analysis results showed that the combination of lymphocytic count along with C-reactive protein levels (LCR) demonstrated the highest correlation with intestinal resection due to intussusception compared with other parameters in the patients, with a sensitivity of 0.82 (0.73–0.86) and specificity of 0.80 (0.57–0.94) for the diagnosis of strangulation.

**Conclusion:**

The preoperative LCR level is a useful marker to predict the need for intestinal resection due to intestinal necrosis in patients with intussusception.

## Introduction

Childhood intussusception is a serious emergent disease that presents with the classic triad of abdominal pain, vomiting and red currant jelly stool [1, 2]. Most intussusception can be managed with nonoperative reduction through fluoroscopic-guided barium enema and pneumatic reduction, and emergency surgery should still be performed for cases in which nonoperative reduction fails [3]. In terms of surgical planning and patient management, it is of great importance to detect potential complications early, as any delay may result in the impairment of intestinal circulation, which can cause intestinal necrosis, perforation or life-threatening secondary peritonitis, resulting in bowel resection and mortality [4].

Based on this understanding, potential biomarkers that can help identify patients with intestinal necrosis should be urgently developed. Systemic inflammation is currently recognized as the hallmark of intestinal necrosis, perforation or secondary peritonitis [5, 6]. Several systemic inflammatory markers, including levels of neutrophils, CRP, albumin, platelets, lymphocytes, and biomarker combination ratios, have been suggested for the early prediction of various inflammatory conditions, such as pancreatitis, acute appendicitis, cancer or mesenteric ischemia [7–10]. Lymphopenia reflects acute inflammation under stress conditions in addition to leukocyte count and neutrophil ratio increases and is often used as a hematological inflammatory marker.

In particular, the combination of inflammatory factors, such as the PLR (platelet-lymphocyte ratio), NLR (neutrophil-lymphocyte ratio), and CAR (CRP-albumin ratio), has been suggested as a valuable inflammatory biomarker [7]. In clinical practice for intussusception patients undergoing surgery, we should address the best combination of these factors in predicting intestinal necrosis in these patients. Furthermore, the cutoff thresholds for these inflammatory markers should be investigated to estimate strangulation in the preoperative period.

In the current research, we performed receiver operating characteristic (ROC) curve analysis to explore the predictive value of different combinations of biomarkers involving the need for bowel resection.

## Materials and methods

### Patient cohort

A retrospective review of a cohort of patients with a diagnosis of intussusception managed with the procedure was performed in a collaborative multidisciplinary program from July 1, 2017, to June 30, 2019. The collaborative institutes included the pediatric general surgical departments at Qingdao Maternity and Child Care Hospital, Yongchuan Hospital of Chongqing Medical University, Jinan Maternity and Child Care Hospital and Chongqing Children’s Hospital. All of these institutes provide tertiary care in their districts with capacities of over 1500 beds. The patients also met the following inclusion criteria: aged > 0.5 years and < 5 years; had failed enema reduction; and had preoperative atropine administered. Subjects were excluded if they were managed directly with surgery. Additionally, we only enrolled patients who underwent open surgery to achieve a more homogenous cohort of patients. The current study protocol was performed following expedited ethical committee approval by the Institutional Review Board of Chongqing Medical University under the protection of personal information.

In clinical practice, the following standard protocol was followed for the management of patients with intussusception. Enema reduction was first attempted under a pressure of 12 kPa, and if reduction was not achieved, the procedure was stopped, and the patient was immediately taken to the operating room for manual surgical reduction or resection with bowel anastomosis. A total of 125 patients who underwent laparotomy were enrolled during the research period, and the patients were divided into two groups according to the presence or absence of intestinal resection: a resection group and a no resection group. Intestinal necrosis was comprehensively evaluated based on the usual practice to obtain blood flow back, which determined resection for the bowel segment.

### Data assessment

The laboratory data on admission collected from the medical records that were obtained within 2 days prior to surgical administration were reviewed. The following inflammation-associated biomarkers were routinely measured during the operation: neutrophils, platelets, CRP, lymphocytes and albumin. We further investigated several combinations of inflammatory markers to identify the marker with the highest accuracy for predicting intestinal resection in patients with intussusception, including the NLR, PLR, LCR and CAR.

### Statistics

The statistical software SPSS (Version 22.0, SPSS Inc., Chicago, IL) was used for the statistical analysis of the data. The continuous data are presented as the means ± standard deviations and medians (interquartile ranges) according to the data distribution and compared using the Mann–Whitney U test, the Shapiro–Wilk test or the Wilcoxon rank-sum test, when applicable. Categorical variables are expressed as frequencies (percentages) and were tested using Fisher’s exact test or the chi-square test.

Receiver operating characteristic (ROC) analysis was conducted to evaluate the various parameters for detecting bowel resection through area under the curve (AUC) calculations. The Youden index in the ROC analysis was carried out to identify the best cutoff points of the different parameters. Statistical significance was set at *p* < 0.05.

## Results

A total of 115 patients within the study period met the previously mentioned inclusion criteria and were included in the final investigation, of which 47 patients (40.9%) were managed with intestinal resection. The baseline features based on intestinal resection are presented in Table 1. As shown in Table 1, the demographic distribution was comparable, including age, sex distribution, and the duration of symptoms. There were significant differences between the two groups regarding the features of covariates for severity, including lesion diameter at admission (*p* = 0.006). There was an increased presentation of red currant jelly stool in the patients with intestinal resection, suggesting a severe strangulation condition (*p* = 0.007). No significant differences were detected in terms of lesion location between the two groups.

**Table 1:** Baseline characteristic of eligible population

	Total Population		
	without intestinal resection(68)	With intestinal resection(47)	*p* Values
Age (yrs), mean ± SD	1.87 ± 0.92	1.79 ± 0.88	0.21
Female: Male	183 (37.5)	191 (39.9)	0.31
Weight (kg), mean ± SD	9.34 ± 2.57	9.22 ± 2.61	0.22
Duration of symptoms on admission (hours), Means±SDs	15.21 ± 11.54	14.76 ± 11.38	0.19
The time to operation after admission (hours), Means±SDs	5.87 ± 2.65	5.38 ± 2.84	0.26
Clinical symptoms, N (%)			
Abdominal pain	35	26	0.42
vomiting	62	46	0.14
red currant jelly stool	31	33	0.007
Location of lesion			
ascending colon	19	11	0.37
hepatic flexure of colon	35	21	0.30
Splenic Flexure of Colon	14	13	0.20
sigmoid colon	0	2	0.17
Diameter of lesion (mm), median (range)	24.6 (20.9–32.8)	28.7 (22.5–39.7)	0.006

To identify the potential of the biomarkers for intestinal necrosis, we explored several parameters that might acutely reflect strangulation conditions, including neutrophils, platelets, CRP, lymphocytes and albumin. Moreover, we identified a few combinations of the above factors, especially up- and downregulation combinations, that might offer more accuracy in predicting intestinal resection in intussusception patients.

As shown in Table 2, intestinal resection was associated with a higher neutrophil count (*p* = 0.013) and CRP level (*p* = 0.002). A similar trend was detected in the combinations of the above parameters, such as the PLR (*p* = 0.008) and NLR (*p* = 0.026). Moreover, anemia was more critical in the patients with intestinal resection (*p* = 0.087), in accordance with a higher proportion of patients needing intraoperative transfusion (data not shown), although statistical significance was not attained.

**Table 2:** Comparison of the preoperative hematologic and biochemical parameters of the two groups

	without intestinal resection(68)	With intestinal resection(47)	*P*
Preoperative Hb level(g/dL), Mean ± SD	11.37 ± 2.66	12.81 ± 3.16	0.087
Albumin(g/L), mean ± SD	39.52 ± 5.68	34.67 ± 6.26	0.102
Lymphocyte(109/L), mean ± SD	1.47 ± 1.1	1.16 ± 0.89	0.064
Neutrophil(109/L), mean ± SD	6.59 ± 3.87	8.78 ± 5.47	0.013
CRP(g/L), mean ± SD	9.42 ± 4.26	12.87 ± 6.83	0.002
Platelet(109/L), mean ± SD	238.3 ± 102.8	276.33 ± 130.15	0.056
PLR, mean ± SD	152.9 ± 127.8	216 ± 184.2	0.008
NLR, mean ± SD	4.64 ± 3.39	6.97 ± 5.11	0.026
LCR, mean ± SD	0.1498 ± 0.0841	0.0963 ± 0.0521	< 0.001
CAR, mean ± SD	0.2561 ± 0.1923	0.3218 ± 0.2123	0.083

*Abbreviations*: *CAR* CRP-albumin ratio, *CRP* C-reactiveprotein, *LCR* lymphocyte–CRP ratio, *NLR* neutrophil–lymphocyte ratio, *PLR* platelet–lymphocyte ratio

The ROC results for all the parameters are shown in Table 3. The area under the curve was found to be statistically significant for CRP levels (*p* = 0.011), the PLR (*p* = 0.008), the LCR (*p* < 0.001), and the NLR (*p* = 0.028) (Table 3), which could significantly indicate intestinal resection with a relatively high accuracy. The highest degree of accuracy was observed for the preoperative LCR at the cutoff value of 0.121 in patients with intussusception, with a sensitivity of 0.82 (0.73–0.86), a specificity of 0.80 (0.57–0.94), and an AUC of 0.81 (0.67–0.93).

**Table 3:** ROC curve results and sensitivity, specifcity values

	CRP	albumin	PLR	LCR	NLR	CAR
AUC (95% CI)	0.71 (0.59–0.84)	0.764 (0.67–0.83)	0.69 (0.48–0.71)	0.81 (0.67–0.93)	0.74 (0.63–0.89)	0.69 (0.51–0.84)
*p* values	0.011	0.083	0.008	< 0.001	0.028	0.064
Cut-off	> 11.26	> 29.4	> 188.5	< 0.121	> 5.72	0.286
Sensitivity (95% CI)	0.68 (0.52–0.81)	0.52 (0.38–0.71)	0.80 (0.57–0.92)	0.82 (0.73–0.86)	0.72 (0.61–0.82)	0.51 (0.36–0.68)
Specifcity (95% CI)	081 (0.72–0.93)	0.83 (0.71–0.88)	0.69 (0.53–0.82)	0.80 (0.57–0.94)	0.83 (0.71–0.96)	0.77 (0.57–0.89)

## Discussion

In the current study, we explored the distinguishing power of combinations of various factors, especially the up and down combinations, detected with preoperative routine laboratory work and delineated several findings. The major finding in this research was that the LCR, defined as the combination of lymphocyte counts along with the CRP levels, was found to be significantly lower in patients who underwent intestinal resection than in patients without resection. Compared with other parameters, such as the neutrophil count, CRP level and NLR level, the LCR was a more reliable indicator of intestinal resection in patients with intussusception.

Intussusception can lead to strangulation, which can cause bowel ischemia and necrosis due to blood flow blockage. The operation for intussusception is highly important to avoid intestinal ischemia and necrosis because a delayed response might lead to unnecessary bowel resection, sepsis, and even death [11, 12]. All these are serious complications following bowel ischemia and necrosis due to intussusception. The primary rationale for early operative intervention is to avoid bowel resection due to ischemia [13]. Previous studies have suggested that approximately one-half of children admitted with intussusception required surgical intervention [13, 14], although conservative management was first attempted for an interval of 5.5 h. In the current research, the incidence of bowel resection was 40.9%, similar to the global data.

To determine the presence of ischemia, there are accumulating studies indicating that various factors are involved in systemic inflammatory conditions. In peripheral blood, as a result of strangulation, measurable inflammatory factors could be detected, which were responses from and released by the local ischemic intestinal wall [15]. In many disease conditions, various inflammatory indicators have been suggested to be valuable in diagnosis and treatment monitoring [16]. However, the best parameters for predicting bowel ischemia and necrosis using peripheral blood systemic inflammatory factor examination in intussusception patients remain unclear.

In the present study, we found that surrogate markers of the severity of the inflammatory response were significantly higher for CRP and leukocyte count values and lower for albumin and lymphocyte levels in patients who underwent intestinal resection than in patients without intestinal resection, indicating an inflammatory response for intestinal ischemia and necrosis. An ROC analysis was further conducted to investigate the optimal accuracy for the involved surrogate markers. In previous research involving inguinal hernias, the LCR was indicated to be higher in patients with strangulation and could be a predictor to indicate bowel resection [5]. In another multivariate analysis, the NLR was also found to be significantly related to hernia strangulation with obvious bowel ischemia [17]. It was concluded that certain surrogate markers can be used to predict intestinal necrosis and have clinical correlations. Furthermore, the LCR has been associated with the prognosis of specific cancer patients, such as stomach cancer and colorectal cancer patients [7, 16, 18]. Similarly, our ROC analysis results showed that among all these parameters, the LCR alone was a good inflammatory parameter and strongly related to intestinal resection. Although the LCR has been researched in many inflammatory conditions, to our knowledge, the use of the LCR in the prediction of intestinal resection was first reported in the current study.

In various clinical settings, including the present study with intestinal resection patients, the LCR value was found to be the most rapid response indicator reflecting systemic inflammatory responses [7]. Here, the LCR value represents the combination of the immunological and inflammatory response with intestinal ischemia due to strangulation during the preoperative period. The negative predictive value comes from the contribution of lymphocytes [19, 20]. Peripheral lymphocytes contribute to the host cytotoxic immune response to intestinal microflora, and the CRP level alone is a good inflammatory marker [21]. As indicated in the current study, a low LCR value means an enhancement of the systemic inflammatory response or impairment of the immunological response in patients with intussusception and could be used to assess intestinal ischemia.

A retrospective analysis reported that the CRP and NLR levels were increased in acute pancreatitis patients [20]. Another study suggested that NLR levels were significantly associated with acute mesenteric ischemia patients who underwent intestinal resection [22, 23]. In the current research, the NLR, CRP level, and neutrophil count were found to be significantly increased in patients who underwent intestinal resection. Among them, the LCR represented the most valuable indicative marker, which is simple and easy to calculate using routine laboratory data without additional techniques or costs.

We acknowledge that the present results must be considered in the context of the study’s limitations, which should be interpreted with caution. The sample size collected in the current research was still somewhat small. Although the study was a single-center investigation, the diagnosis of intestinal necrosis and the decision to perform resection depended on the opinions of individual surgeons, which are subjective, with the possibility of over- or underclassification of the degree of intestinal necrosis. To conduct a powerful study that is specifically aimed at intestinal necrosis in patients with intussusception, further investigation with more patients is needed to validate the role of the preoperative LCR, with a consistent cutoff value in patients undergoing intestinal resection.

In conclusion, the current research suggested that the preoperative LCR from routine hematological parameters was a promising predictive factor for bowel resection in patients with intussusception, which should also be effective for perioperative management. To date, no other study has addressed this issue specifically in pediatric patients with appendicitis.

## Data Availability

The datasets used and/or analyzed during the current study are available from the corresponding author on reasonable request.
